# Urachal carcinoma: a large retrospective multicentric study from the French Genito-Urinary Tumor Group

**DOI:** 10.3389/fonc.2023.1110003

**Published:** 2023-01-19

**Authors:** M. Guerin, C. Miran, E. Colomba, M. Cabart, T. Herrmann, S. Pericart, D. Maillet, Y. Neuzillet, A. Deleuze, E. Coquan, M. Laramas, C. Thibault, B. Abbar, B. Mesnard, D. Borchiellini, C. Dumont, E. Boughalem, JL. Deville, M. Cancel, C. Saldana, A. Khalil, G. Baciarello, A. Flechon, J. Walz, G. Gravis

**Affiliations:** ^1^ Department of Medical Oncology, Institut Paoli-Calmettes, Marseille, France; ^2^ Department of Medical Oncology, Centre Leon-Berard, Lyon, France; ^3^ Department of Cancer Medicine, Institut Gustave-Roussy, University of Paris Saclay, Villejuif, France; ^4^ Department of Medical Oncology, Institut Bergonie, Bordeaux, France; ^5^ Department of Medical Oncology, Centre Jean-Perrin, Clermont-Ferrand, France; ^6^ Department of Anatomo-pathology, Institut Universitaire du Cancer, Centre Hospital-Universitaire de Toulouse, Toulouse, France; ^7^ Department of Medical Oncology, Centre hospitalo-Universitaire Hospices civils, Lyon, France; ^8^ Department of Urology, Hopital Foch, Paris, France; ^9^ Department of Medical Oncology, Centre Eugene Marquis, Rennes, France; ^10^ Department of Medical Oncology, Centre François Baclesse, Caen, France; ^11^ Department of Medical Oncology, Centre Hospitalo-Universitaire, Grenoble, France; ^12^ Department of Medical Oncology, Hopital Europeen Georges Pompidou, Paris, France; ^13^ Department of Medical Oncology, Hopital Pitié-Salpetriere, Paris, France; ^14^ Department of Urology, Centre Hospitalo-Universitaire, Nantes, France; ^15^ Department of Medical Oncology, Centre Lacassagne, Nice, France; ^16^ Department of Medical Oncology, Hopital Saint-Louis, Paris, France; ^17^ Department of Medical Oncology, Centre Paul Papin, Angers, France; ^18^ Department of Medical Oncology, Centre Hospitalo-Universitaire Timone, Marseille, France; ^19^ Department of Medical Oncology, Centre Hospitalo-Universitaire Bretonneau, Tours, France; ^20^ Department of Medical Oncology, Hopital Henri Mondor, Paris, France; ^21^ Department of Medical Oncology, Hopital tenon, Paris, France; ^22^ Department of Urology, Institut Paoli-Calmettes, Marseille, France

**Keywords:** retrospective, urachal cancer, multicentric, urachus, rare disease

## Abstract

**Introduction:**

Urachal cancer (UrC) is a rare, non-urothelial malignancy. Its natural history and management are poorly understood. Although localized to the bladder dome, the most common histological subtype of UrC is adenocarcinoma. UrC develops from an embryonic remnant, and is frequently diagnosed in advanced stage with poor prognosis. The treatment is not standardized, and based only on case reports and small series. This large retrospective multicentric study was conducted by the French Genito-Urinary Tumor Group to gain a better understanding of UrC.

**Material and Methods:**

data has been collected retrospectively on 97 patients treated at 22 French Cancer Centers between 1996 and 2020.

**Results:**

The median follow-up was 59 months (range 44-96). The median age at diagnosis was 53 years (range 20-86), 45% were females and 23% had tobacco exposure. For patients with localized disease (Mayo I-II, n=46) and with lymph-node invasion (Mayo III, n=13) median progression-free-survival (mPFS) was 31 months (95% CI: 20-67) and 7 months (95% CI: 6-not reached (NR)), and median overall survival (mOS) was 73 months (95% CI: 57-NR) and 22 months (95% CI: 21-NR) respectively. For 45 patients with Mayo I-III had secondary metastatic progression, and 20 patients were metastatic at diagnosis. Metastatic localization was peritoneal for 54% of patients. Most patients with localized tumor were treated with partial cystectomy, with mPFS of 20 months (95% CI: 14-49), and only 12 patients received adjuvant therapy. Metastatic patients (Mayo IV) had a mOS of 23 months (95% CI: 19-33) and 69% received a platin-fluorouracil combination treatment.

**Conclusion:**

UrC is a rare tumor of the bladder where patients are younger with a higher number of females, and a lower tobacco exposure than in standard urothelial carcinoma. For localized tumor, partial cystectomy is recommended. The mOS and mPFS were low, notably for patients with lymph node invasion. For metastatic patients the prognosis is poor and standard therapy is not well-defined. Further clinical and biological knowledge are needed.

## 1 Introduction

First described in 1863 (1), urachal cancer (UrC) is a rare, non-urothelial malignancy that represents less than 1% of all bladder cancers (2, 3), and 10% of all adenocarcinomas of the urinary bladder. UrC develops in the urachus, an embryonic remnant of the urogenital sinus and allantois, which connects the dome of the bladder to the umbilicus. This transitory structure appears on the 28^th^ day of embryonic development, and at birth, only a thin fibrous ligament remains. Although rare, embryonic vestiges may persist, and from these, malignant abnormalities can develop in adulthood (4, 5). The urachal remnant is generally localized to the bladder dome, but UrC displays distinct pathologic and clinical features. For diagnosis, UrC must be distinguished from urothelial adenocarcinomas, which arise from any portion of the urothelium. Moreover, UrC and urothelial adenocarcinomas are associated with different patient demographics and tumor characteristics (6). Therefore, these two tumors require different management and survival strategies. The most common clinical presentation of UrC is macroscopic hematuria or abdominal pain, but currently, no risk factors have been identified. To date, no consensus has been reached for the diagnosis of UrC, and most clinicians apply the criteria proposed by Sheldon: (*i*) a tumor located in the dome or anterior wall of the bladder, (*ii*) absence of intestinal metaplasia or cystitis glandularis beyond the dome, (*iii*) absence of urothelial carcinoma (*iv*) exclusion of a primary adenocarcinoma of different origin; and (*v*) the presence of calcification in a midline abdominal wall mass, which is nearly pathognomonic of UrC (7, 8). The most common histological type of UrC is an adenocarcinoma that produces abundant mucin; thus, several studies have used glandular differentiation as a criterion for diagnosis (3, 7, 9). Some authors have highlighted diagnostic features in tumor sections, including strong, diffuse CK20 expression, frequent CDX-2 expression, CK7 expression in about half the samples (10), and no GATA-3 expression (3, 11). However, the rarity of UrC has led to a limited number of published studies on clinico-pathologic series.

In 1984, Sheldon et al., were the first to propose an anatomic stratification; UrC was separated into 8 categories, based on 117 patients described in the English literature (7). Later, the Mayo clinic evaluated over 50 years of records on 66 patients with UrC (9). Based on those results, they defined another stratification that was less complex, but highly correlated with that proposed by Sheldon et al. The Mayo stratification described 4 distinct stages, as follows: stage I: tumors confined to the urachus/bladder; stage II: tumors that extend beyond the muscular layer of the urachus/bladder; stage III: tumors that infiltrate the regional lymph nodes; and stage IV: tumors that infiltrate non-regional lymph nodes or distant sites. In 2006, Pinthus et al. described a Canadian cohort of 40 patients with UrC that had been treated from 1976 to 2003 (12). They graded the tumors as follows: well differentiated tumors (grade I), which were associated with better disease-specific survival (90% at 5 years); moderately differentiated tumors (grade II); and poorly differentiated tumors (grade III), which were associated with 100% mortality at 5 years.

For localized disease, the standard of care is a partial cystectomy, which is associated with a median overall survival (OS) of around 10 years (9). Furthermore, a pelvic lymphadenectomy is typically offered, but the data are controversial and, in the current literature, a benefit to OS has not been formally demonstrated (13, 14). Survival was also correlated with the pathological stage, the surgical margins, the presence of lymphovascular invasion, and omphalectomy (15), which underlines the importance of a complete resection in UrC. Few data are available about neoadjuvant or adjuvant therapy, and those studies showed no significant improvement in survival (16).

Unfortunately, because UrC develops in a silent area, it is typically diagnosed in an advanced stage and cannot be treated with surgery. Due to its rarity and aggressiveness, the prognosis is poor. The standard of care in this setting is not well defined; it is mainly based on case reports and small series. Several regimens of chemotherapy are typically administered, and the median OS is 1.3 years (17). The largest series of patients with advanced UrC was described by a group at the M.D Anderson Cancer Center in 2003. That study included 26 patients, and of those, 20 received chemotherapy. Most responses were obtained with regimens of cisplatin and 5-fluorouracil (FU); others responded to regimens based on a combination of methotrexate, vinblastine, doxorubicin, and cisplatin (MVAC), Paclitaxel, or Ifosfamide (18).

The present large, retrospective multicentric study from the French Genito-Urinary Tumor Group (GETUG) aimed to gain a better understanding of UrC by studying 97 patients with UrC.

## 2 Materials and methods

Data were retrospectively collected from 22 French institutions with expertise in onco-urology (comprehensive cancer centers and public hospitals) affiliated with GETUG. We retrieved data on patients diagnosed with UrC from 1996 to 2020. All patients were included when they had UrC, defined by each center, based on histological, radiological, and/or cystoscopic criteria, and were aged ≥18 years at diagnosis. We applied no exclusion criteria. Clinical and biological data were collected from the medical charts, including baseline characteristics, pathologic findings, treatments administered, and follow-up. The UrC diagnosis was performed by a pathologist at each institution, for the majority on primary tumors. In some cases, diagnoses were reviewed by an expert pathologist. The stage at diagnosis was assessed with the Mayo clinic classification system. Patients were staged as one of four stages: I= tumors confined to the urachus/bladder; II=tumors that extended beyond the muscular layer of the urachus/bladder; III=tumors that infiltrated the regional lymph nodes; or IV=tumors that infiltrated non-regional lymph nodes or distant sites.

Response to therapy was assessed based on computed tomography scans and RECIST criteria. According to the evaluation performed at the local centers, responses were recorded as a partial or complete response, stable disease, and progression.

Descriptive and survival analyses were performed with R software version 3.6.3. Survival values were estimated with the Kaplan-Meier method. Prognostic factors were analyzed with a Cox model. This study was authorized by the Institutional Review Board of the Institut Paoli-Calmettes, Marseille, France. Molecular analyses were assessed using polymerase chain reaction or Next Generation Sequencing (NGS) (targeted or whole exome).

## 3 Results

### 3.1 Patients

**Figure 1:**
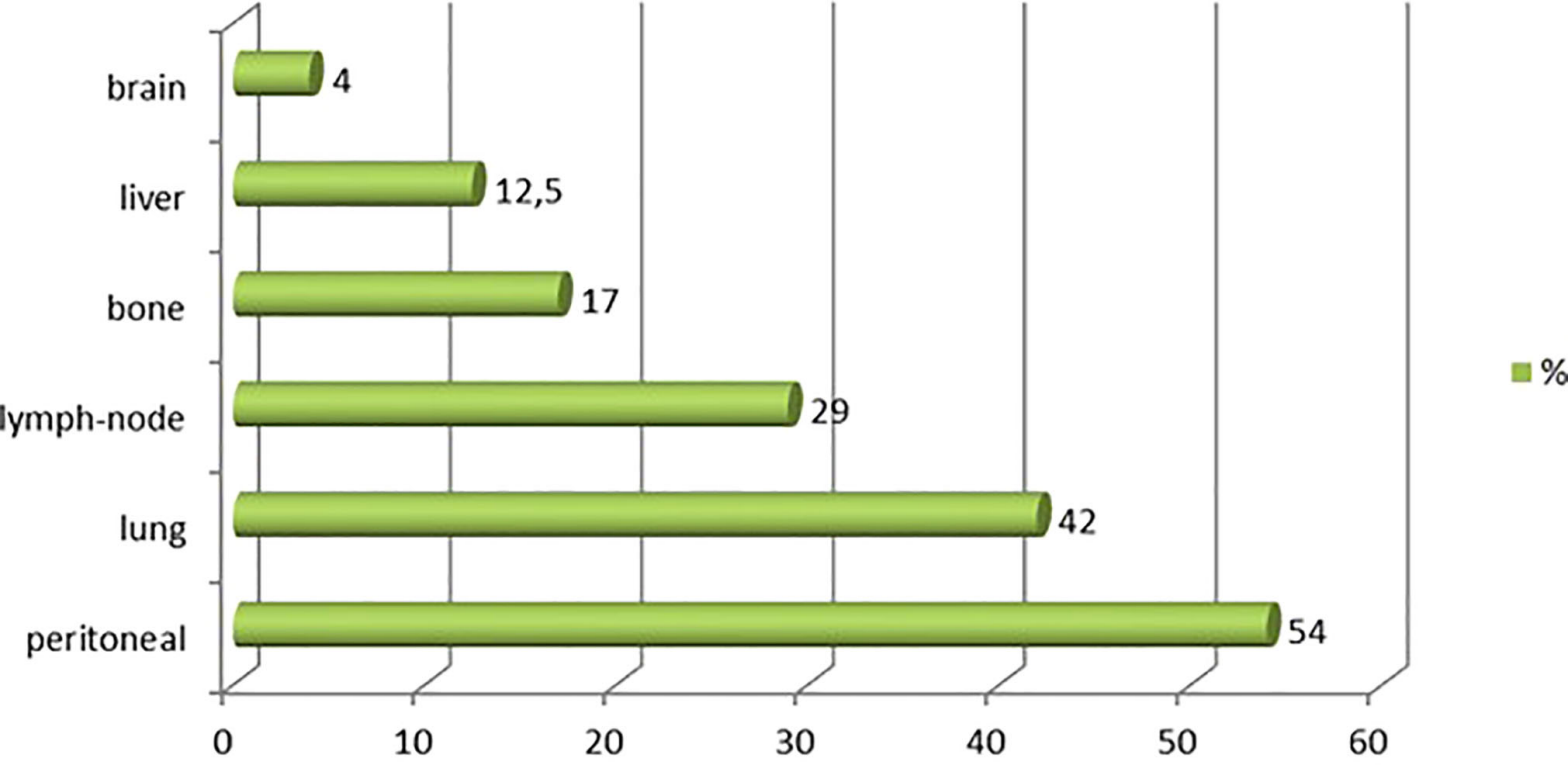
Metastatic sites observed for urachal carcinoma. Chart shows the distribution of metastatic sites among 97 patients with urachal cancer.

Between 1996 and 2020, 97 patients were treated for UrC at the 22 studied French cancer centers. The median follow-up was 59 months (range 44 to 96). Most patients were male (55%), the median age was 53 years at diagnosis (range 20-86), and the predominant performance status was 0-1. Patient characteristics are shown in **Table 1**. Cardiovascular comorbidities were present in 47% of patients, and 59% were overweight (n=23). Only 39% (n=31) of patients had a history of tobacco consumption; of these, 19 patients (23%) had consumed more than 15 pack-years (range: 20-50 pack-years). No patient reported a congenital urachus malformation in childhood. The most common symptoms that led to the diagnosis were hematuria (63%) and/or abdominal pain (18%). In 55 patients (57%), a typical mass in the bladder dome was clearly described on cystoscopy. In 42 patients, the diagnosis was based more on histological and/or radiological criteria. At diagnosis, 76 patients (79%) had non-metastatic disease: the tumor was localized in 46 patients and it was locally advanced in 13 patients (17 patients had missing data on lymph node invasion). Twenty patients (21%) had *de novo* metastatic disease. Among the 76 patients with non-metastatic disease at diagnosis, 45 experienced a metastatic evolution (59%; n=8 with missing data). Among the 65 patients with metastatic disease, the most common metastatic site was the peritoneum (54% peritoneal carcinomatosis). Other metastatic sites were the lung (42%), distant lymph nodes (29%), bone (17%), liver (12.5%), and brain (4%; **Figure 1**).

**Table 1:** Patient characteristics.

Baseline characteristics	N=97	%
Median age, years (range)	53	(20-86)
Age ≤55 years	53	55
Age >55 years	44	45
Sex		
Male	53	55
Female	44	45
Tobacco		
Yes	31	39
no	49	61
Alcohol		
Yes	3	4
no	73	96
Performance Status		
0-1	86	94
≥2	5	6
Cardiovascular Comorbidities		
Yes	42	47
no	48	53
Diabetes		
Yes	9	10
no	80	90
High blood pressure		
Yes	19	21
no	70	79
Dyslipidemia		
Yes	12	14
no	77	86
Overweight BMI≥25 kg/m^2^		
Yes	23	59
no	16	41
Initial symptoms	83/91	91
Hematuria		
Yes	57	63
no	33	37
Abdominal pain		
Yes	16	18
no	74	82
Stage (Mayo classification)		
Non metastatic (Stage 1 to 3)	76	79
*Stages 1-2*	*46*	*49*
*Stage 3*	*13*	*13*
Metastatic Stage 4	20	21

Values are the number and percentage, unless otherwise indicated.

**Figure 2:**
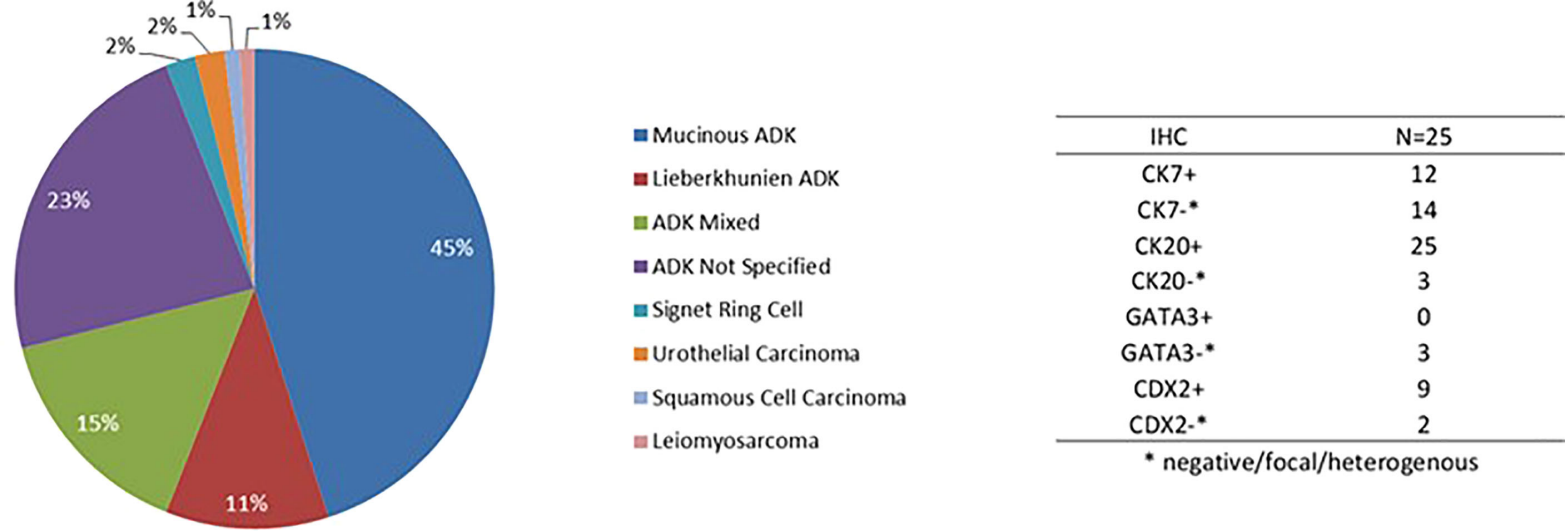
Histologic subtypes of urachal cancer. ADK, adenocarcinoma; IHC, immunohistochemistry result.

The histological subtypes observed are summarized in **Figure 2**. The UrC diagnosis was reviewed by an expert pathologist for 16 patients. Adenocarcinoma was the most common pathological subtype (94%). Adenocarcinomas were classified as: mucinous (45%), Lieberkuhnian (11%), mixed (15%), or non-specified (23%). Other subtypes included signet-ring cell (2%), urothelial carcinoma (2%), squamous-cell carcinoma (1%), and leiomyosarcoma (1%). Immunohistochemistry data were available for 28 patients. Of the 28 UrC samples, 25 stained positive and 3 stained negative for CK20, and 12 stained positive and 14 stained negative for CK7 (**Figure 2**).

### 3.2 Treatment and prognosis for localized tumors

Among the 76 patients with localized or locally-advanced tumors at diagnosis, 69 underwent a cystectomy (n=7 with missing data). Of these, 55 patients underwent a partial cystectomy, including 24 patients that underwent a loco-regional lymphadenectomy; and 14 patients underwent a radical cystectomy, including 8 patients that underwent a lymphadenectomy. The median metastatic progression-free survival (mPFS) was similar between groups: 20 months (95% confidence interval [95% CI]: 14-49) vs. 18 months (95% CI: 11-Not Reached (NR)), respectively (*p=*0.9). Among the 69 patients that underwent a cystectomy, according to the Mayo classification, 46 patients had stages I-II disease, 11 patients had T1-T2 tumors, and 31 patients had T3-T4 tumors (n=4 with missing data). Thirteen patients had radiological (n=4) or histological (n=9) lymph-node invasions. Vascular or peri-nervous emboli occurred in 18 patients. Twelve patients had incomplete resections, and 32 patients had an omphalectomy.

**Figure 3:**
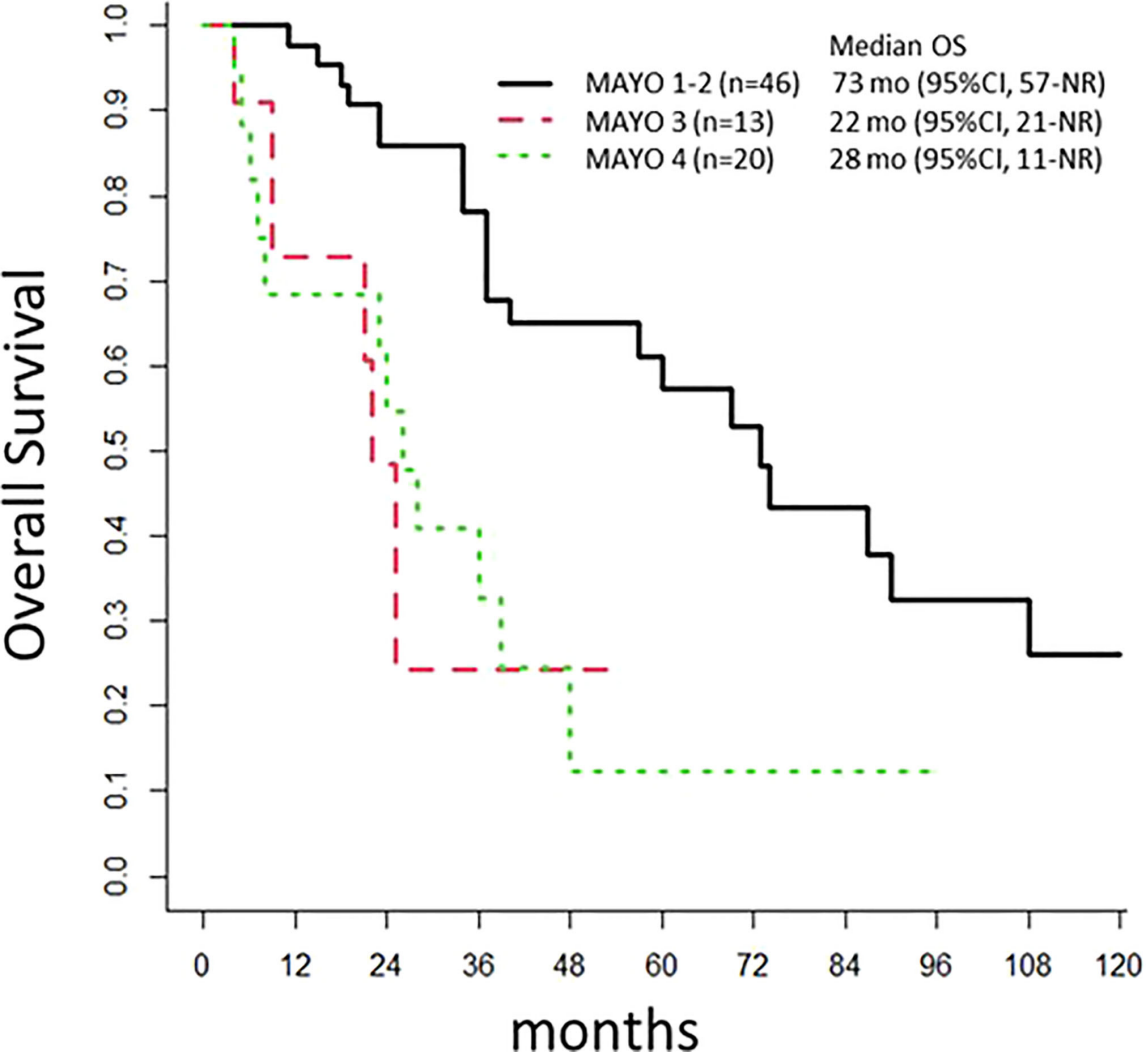
Overall Survival. The population of 97 patients with urachal cancer at diagnosis is stratified according to the Mayo classification. OS, overall survival; mo, months; NR, Not Reached.

Patients with lymph node invasions (Mayo class III) had a poor prognosis, with an mPFS of 7 months (95% CI: 6-NR) and an mOS of 22 months (95% CI: 21-NR). Patients with Mayo I-II disease had an mPFS of 31 months (95% CI: 20-67) and an OS of 73 months (95% CI: 57-NR; *p*<0.001; **Figure 3**). The mPFS was 29 months (95% CI: 17-NR), for patients that underwent an omphalectomy, and 17 months (95% CI: 6-59) for those that did not require an omphalectomy (*p=*0.2). The mPFS was 18.5 months (95% CI: 7-NR), for patients with positive margins, and 21 months (n=46, 95% CI: 12-56) for patients with a complete resection (*p=*0.6). Among 69 patients treated with a cystectomy, 11 had a loco-regional relapse; of these, 8 underwent a secondary surgery, and 45 experienced metastatic progression.

Sixteen patients received adjuvant therapy; of these, 5 received radiotherapy and 12 received radiotherapy and/or chemotherapy (n=12). The majority (n=8) received a FU-platin based combination. Other chemotherapy protocols included gemcitabine-platin (n=1), MVAC (n=1), and irinotecan-FU (n=1). A majority of the patients that received chemotherapy had a high tumor burden; 6 patients had positive lymph nodes, and the mPFS was 13.5 months (95% CI: 8-NR). Among the patients that received adjuvant radiotherapy, 3 had incomplete resections and 2 had positive lymph nodes (n=3 with missing data); the mPFS was 14 months (95% CI: 7-NR).

The median duration between the UrC diagnosis and metastatic disease was 19 months (95% CI: 12-40).

### 3.3 Treatment and prognosis for metastatic disease

Among the 65 patients with metastatic disease, 44 died of UrC. The mOS was 23 months (95% CI: 19-33) for those diagnosed with metastatic disease, 28 months (95% CI: 11-NR) for those with *de novo* metastatic disease, and 22 months (95% CI: 17–29) for those with metastatic progression after local treatment (*p=*0.47). Fifty-seven patients received chemotherapy (n=2 with missing data), and most received the first-line chemotherapy administered for colorectal cancer. Indeed, most patients received platinum-based chemotherapy (**Table 2**), including a platin-FU combination (n=38 patients), a platin-gemcitabine combination (n=8 patients), and a platin-anthracycline combination (n=3 patients); only 6 patients received irinotecan and/or FU without platinum.

**Table 2:** Treatments and survival for patients with metastatic disease.

Chemotherapy regimen	Best Response	Best Response	mPFS, months (95% CI)	mOS, months (95% CI)
FU-based combined with platin* (n=38)	Progression	9	5 (3-11)	48 (19-NR)
	Stable disease	8		
	Response	15 (3 complete response, 1 dissociated response)		
FU-based without Platin (n=5)	Progression	1	4 (3-NR)	19 (19-NR)
	Stable disease	2		
	Response	2 (1 complete response)		
Platin-gemcitabine (n=8)	Progression	4	2 (0-NR)	20 (8-NR)
	Stable disease	0		
	Response	2		
Platin-Anthracycline (n=3)	Progression	1	11 (2-NR)	26 (0-NR)
	Stable disease	1		
	Response	1		
Cisplatin** (n=14)	Progression	4	9 (2-NR)	20 (10-NR)
	Stable disease	2		
	Response	7		

*cisplatin (n=6) or oxaliplatin (n=32).

**Median PFS was 4.5 months (95%CI: 3-12; p=0.8); mOS 24 months (95% CI: 21-48; p=0.08) without platinum.

In combination with chemotherapy, 11 patients received bevacizumab, and 6 patients received anti- endothelial growth factor receptor (anti-EGFR) monoclonal antibodies in the first- or second-line treatment. The median OS times were 19 months (95% CI: 7-NA), for those that received anti-EGFR, and 39 months (95% CI: 17-NR) for those that received bevacizumab.

Few patients (n=22) had data available from molecular analyses, based on the polymerase chain reaction (n=6) or NGS (targeted sequencing: n=10; whole exome sequencing: n=6). The most frequent mutation observed was in the *KRAS* gene (G12V, G13D; n=5 patients).

## 4 Discussion

Urachal cancer is a rare tumor, which lacks a standard of care for managing local or advanced/metastatic disease. The present study was one of the largest retrospective series of UrC, particularly for a study that focused on metastasis.

In 2016, a meta-analysis of 1010 patients with UrC was conducted that included 24 studies identified with a PubMed search (19). Two other studies by Wright et al. and Bruins et al. described the largest series, with 151 and 152 patients, respectively (6, 16). When all the data from these studies were combined, they included chemotherapy data from 74 patients with UrC, with numerous missing data. Moreover, due to the large number of various chemotherapy combinations, those authors did not account for the use of other agents added to cisplatin or FU. Two other studies by Flammia et al. (2021) and Mylonas et al. (2017) searched the SEER database and collected data on 274 and 420 patients, respectively, but they only reported incidence and survival results (20, 21).

Although UrC is localized to the bladder dome, its biological and histological characteristics are more similar to colorectal carcinomas than to other urothelial cancers. For example, UrC affects younger adults, with a median age at diagnosis of 53 years (3, 16, 22). In comparison, the median age of patients with urothelial carcinomas is 73 years (23). UrC also seems to be different from urothelial carcinoma in its presentation. UrC affects more women than urothelial carcinomas [50% vs. 25% (24)], and only 23% of patients with UrC had a significant tobacco history, which is the leading cause of bladder carcinoma (it accounts for 50%-65% of new cases annually) (23). Interestingly, urachal malformations were reported to be a risk factor for UrC development; however, in our study, no patient reported this type of history in childhood (4). Although the most common clinical presentation of UrC was macroscopic hematuria or abdominal pain, similar to bladder carcinomas, UrC has a different histopathology and prognosis (6, 25). Similar to other studies, our study found that the most frequent subtype was adenocarcinoma, and the majority exhibited mucin production; thus, mucosuria should be systematically reported, when UrC is suspected (10).

Our data were consistent those reported in Reis’ review. They found that 89% (n=25) of UrC samples strongly and diffusely expressed CK20, and CK7 was expressed in 54% of samples (n=14) (10); CDX-2 was expressed in 9 of 11 samples; GATA-3 was analyzed in only 3 samples, and the staining was negative for all 3 (3, 11). Finally, because the urachus is a small remnant, UrC was poorly defined in all the previous retrospective studies, which led to a misestimation of UrC prevalence (21). In the present study, a bladder dome mass was described on cystoscopy in only 55 patients; for the other patients, the diagnosis was based on histological and/or radiological criteria. To date, except for the criteria proposed by Sheldon (7), there is no consensus of criteria for clearly defining tumors of the urachus. Therefore, digestive endoscopies should be systematically discussed, and a centralized anatomo-pathological and radiological review by an expert should be conducted, when possible.

For localized tumors, some consistent data favor treatment with a partial cystectomy. These data include the series described by Bruins (16) in 2012, which included 152 patients from the Netherlands diagnosed with UrC between 1989 and 2009. Of those patients, 77% underwent a partial or radical cystectomy, but neither treatment showed a superior outcome, and the median OS was 48 months. More recently, Yu et al. published a retrospective study that included 203 patients with UrC, including 82% that had localized tumors. That study also found no difference in outcomes between the partial and radical cystectomy (13). In the study by Yu et al, although a lymphadenectomy (23%) was not correlated with the UrC prognosis, positive lymph-nodes (11%), positive margins (8.4%), and an en-bloc resection of the umbilicus (5.9%) were identified as prognostic factors that were correlated with survival. In the present study, 80% of patients with non-metastatic tumors underwent a partial cystectomy, and the mPFS was 20 months, similar to the mPFS for a complete cystectomy. One important prognostic factor was the lymph-node status: patients with negative lymph nodes had an mOS of 73 months, clearly superior to the mOS of 22 months observed for patients with positive lymph nodes, and similar to the mOS observed in the metastatic cohort. Therefore, these results suggested that induction chemotherapy should be discussed for patients with positive radiologic lymph-nodes. Additionally, we noticed a trend towards omphalectomy as a prognostic factor, but it did not reach significance: the mPFS was 29 months for those with an omphalectomy, compared to 17 months for those without an omphalectomy (*p=*0.2). These results were consistent with those from previous studies (9, 13, 15, 16). Thus, an omphalectomy should be considered a standard procedure for patients with localized disease.

Although a partial cystectomy is currently a standard procedure in managing UrC, few data are available to support peri-operative radiotherapy and/or chemotherapy. Yu et al. reported the most consistent data, with 64 patients that received adjuvant chemotherapy. However, those patients showed no improvement in survival over patients that did not receive adjuvant chemotherapy (13). In that study, cisplatin-FU (34%) and cisplatin-gemcitabine (22%) were the chemotherapies most commonly administered. In our study, only 16 patients received peri-operative chemo- or radiotherapy, and these treatments were not associated with a better outcome. However, the lack of improvement could probably be explained by the fact that most of those patients had invasive UrC with positive lymph nodes. Although an incomplete resection was not identified as a prognostic factor in our study, previous studies emphasized that positive margins were associated with worse survival; therefore, neo-adjuvant chemotherapy should be an issue of debate for this population (9, 13, 16).

The most frequent metastatic site for UrC is the peritoneum (58% in our study). However, this location is difficult to assess with a conventional computed tomography scan. Thus, fluorodeoxyglucose-positron emission tomography (FDG-PET) and MRI, with higher sensitivity and specificity (26–28), could be discussed before a cystectomy to improve the selection of patients and evaluate the potential indication of first-line chemotherapy (16, 26). Because the peritoneum is frequently affected, hyperthermic intraperitoneal chemotherapy (HIPEC) could also be investigated as a peri-operative treatment (30). In our study, only one patient with *de novo* metastasis received a partial cystectomy combined with HIPEC, and that patient survived more than 3 years. The other preferred metastatic sites for this tumor were the lung (44%), non-loco-regional lymph nodes (33%), bone (18%), and liver (18%), consistent with previous studies (31).

The mOS was poor in this young population; our mOS results were 28 months for patients with *de novo* metastatic disease and 22 months for patients with metastatic relapse (6). Due to the scarcity of data, there is no standard therapy for metastatic disease. In 2003, Siefer-Radtke performed a retrospective study of 42 patients, and of those, 26 patients developed metastases. Twenty patients received chemotherapy, and the mOS was 20 months (18). Flammia et al. (2021) evaluated the benefit of chemotherapy in 274 patients with metastatic UrC, between 2014 and 2016 in Europe and the USA. In that study, the population was slightly different from our population; the median age at diagnosis was 70 years, and 66% were male. Among that metastatic population, only 32% received chemotherapy, and 12% underwent a cystectomy. They reported a worse prognosis than those reported in previous studies, with an mOS of 6 months. However, the median OS was significantly better for patients that received chemotherapy (17 months) than for patients that were chemotherapy-naïve (2 months). More surprisingly, their results suggested that, in that metastatic population, a cystectomy improved the OS; the mOS was 5 months without, and 31 months with a cystectomy (*p=*0.001). However, those authors probably underestimated the true prevalence of UrC, due to the slightly different population (i.e., the different median age at diagnosis and male percentage) compared to others studies, the lack of data on chemotherapy regimens, and the numerous missing information (vital parameters, performance status, burden of metastatic tumor). Therefore, those results should be interpreted with caution (21). Szarvas et al. (2016) performed a meta-analysis of 24 studies. They described 74 patients with metastases that were treated with chemotherapy. They compared the radiographic response of patients treated with cisplatin-based therapy (n=22), FU-based therapy (n=16), combined cisplatin + FU therapy (n=14), and other therapies (n=22). The cisplatin-based combination was associated with few responses (9%, n=2) and 45% (n=10) stable disease. The most effective treatment seemed to be the cisplatin + FU combination, with a 43% response rate (n=6), 43% (n=6) stable disease, and only 14% (n=2) progression, compared to 31% (n=5) progression for the FU alone group (19). Chen et al. evaluated the treatment outcome of chemotherapy in 24 patients with relapsed or metastatic UrC. Patients that received platinum had better outcomes, with an mPFS of 8 months, compared to 3.8 months for those that did not receive platinum (*p=*0.0032), but the mOS results were not significantly different between groups (29 months vs. 16 months, respectively; *p=*0.63). No significant differences in mOS or mPFS were observed for patients treated with or without FU and for patients treated with or without paclitaxel. Seven patients had next-generation sequencing (NGS) data; of those, 5 patients carried *TP53* mutations, but no information was reported on *RAS* mutations (31).

In our retrospective study, of the 65 patients with metastatic disease, 57 received chemotherapy. The majority (n=38) were treated with the platin + FU combination, and the mPFS was 6 months (95% CI: 3-11), but no benefit was observed in OS, compared to other regimens. The benefit of platinum was not clear, but the majority of patients (n=49) received this drug as a first-line treatment, and 3 out of 6 other patients received this drug as a second- or third-line treatment. Although cisplatin seemed to provide a better mPFS (9 months with vs. 4.5 months without cisplatin), the difference was not significant (*p=*0.8), and cisplatin did not improve the mOS.

Recently, Loizzo et al. published an evidence-based guide for clinical practice (8). For the metastatic stage, despite few data, they proposed chemotherapy based on FU and either cisplatin or oxaliplatin. Our study highlighted the modest benefit provided with these combinations; we observed similar survival results among patient treated with the different combinations. Alternatively, targeted therapies might be more interesting: patients (n=11) treated with bevacizumab survived longer (39 months). Indeed, one case was reported in 2015, where a patient with a partially necrotic tumor at biopsy achieved stable disease after a 2nd-line treatment of 8 cycles of FOLFIRI-bevacizumab (32). A few studies also reported prolonged survival with anti-EGFR treatment. In our study, only 6 patients received anti-EGFR monoclonal antibodies (33).

To improve our understanding of this rare disease and its outcome, NGS should be performed, and patients should be included in clinical basket trials as much as possible. In our study, only 22 patients benefited from NGS analyses; no tumor exhibited microsatellite instability (MSI), and the most frequent mutation (n=5 patients) was in the *KRAS* gene (G12V and G13D). This mutation is frequently found in colorectal carcinomas (34, 35). In 2016, Behrendt et al. published a review of literature about genetics and biological markers, mostly derived from case reports or cohort studies. They highlighted mutations of HER2 (20%), KRAS (20%) and GNAS (10%) (36). In 2018, Reis et al. found pathogenic gene alterations in 70 patients with UrC, including 73 pathogenic gene mutations and 4 gene amplifications (37). The most common gene mutations were detected in *KRAS*/*NRAS*, *BRAF*, or *PIK3CA*, and 16 patients had concomitant *TP53* gene mutations. *FGFR-3* mutations were not detected in any patient. Analyses of MSI were performed in 56 samples, and only one was classified as MSH-2; the others were not MSI. Sirintrapun et al. found controversial results; they reported that MSI status was detected in 3 patients of 7 (38). Cornejo et al. in 2020 evaluated 36 UrC and 4 bladder adenocarcinomas using targeted NGS of 50 cancer “hotspot” mutations. They found similar alterations than previously described with distinct mutation profiles between UrC and bladder adenocarcinoma, but no differences between the different histologic subtypes of UrC. MSI was identified in 82% of UrC (39).

In conclusion, UrC is a rare disease, with a poor prognosis. We lack knowledge of UrC and specific guidelines for treatment. Patients with localized tumors have a good prognosis. Conversely, when patients have lymph-node invasion, the prognosis is similar to that observed in the metastatic population. Patients eligible for a cystectomy should be evaluated better, with FDG-PET or MRI. In metastatic stages, most patients received a platinum-based chemotherapy, but none of the platinum-based regimens significantly improved patient survival. Molecular characteristics need to be investigated, and clinical trials with targeted therapy should be conducted. Due to the rarity of the patients with UrC, they should be treated at expert centers, and they should undergo pathological and radiological reviews.

## Data availability statement

The raw data supporting the conclusions of this article will be made available by the authors, without undue reservation.

## Ethics statement

The studies involving human participants were reviewed and approved by Institut Paoli-Calmettes. Written informed consent for participation was not required for this study in accordance with the national legislation and the institutional requirements.

## Author contributions

MG and GG contributed to conception and design of the study. MG organized the database and performed the statistical analysis. MG and GG wrote the first draft of the manuscript. All authors contributed to manuscript revision, read, and approved the submitted version.
